# Supplementary material for: Characterization of Novel SARM1 Inhibitors for the Treatment of Chemotherapy-Induced Peripheral Neuropathy
Source: Biomedicines. 2024 Sep 18;12(9):2123. doi: 10.3390/biomedicines12092123 (PMC11428815; doi:10.3390/biomedicines12092123)
Supplement: Supplementary file 1 [file biomedicines-12-02123-s001.zip › biomedicines-3171848-supplementary.pdf]

Table S1. Structure of 6 compounds and the IC_{50}.

cpd	tri-cyclic fused ring A	PC6 IC_{50}
174		17.2 nM
333P1		189.3 nM
160	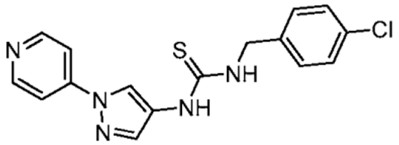	21.4 nM
60	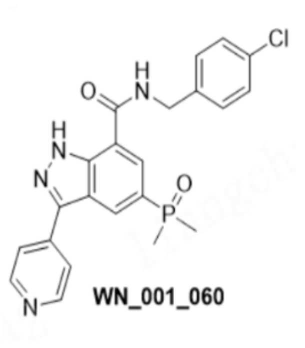	56.3 nM

204	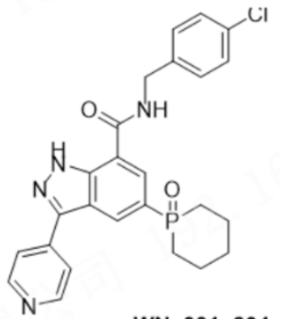 WN_001_204	56.8 nM
192	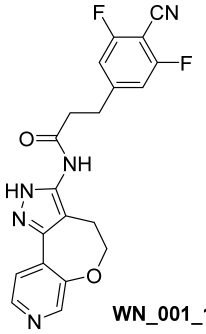 WN_001_192	33.6 nm

Supplementary Figure S1, Protection of axons from axotomy when administrated with 6 compounds.

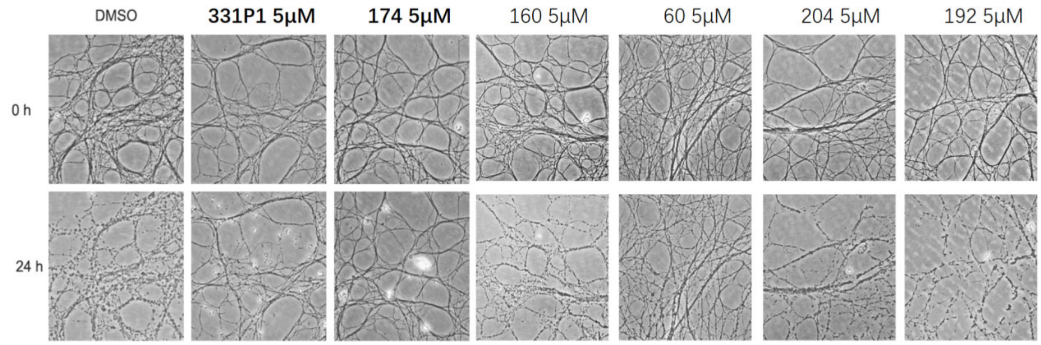

Figure S1. Axons from DRG neurons were axotomized, and the degeneration of distal axons monitored over concentration of selective 6 compounds.

Supplementary Figure S2, Protection of PAC-injured axons when administrated with compounds Nura and 331 P1.

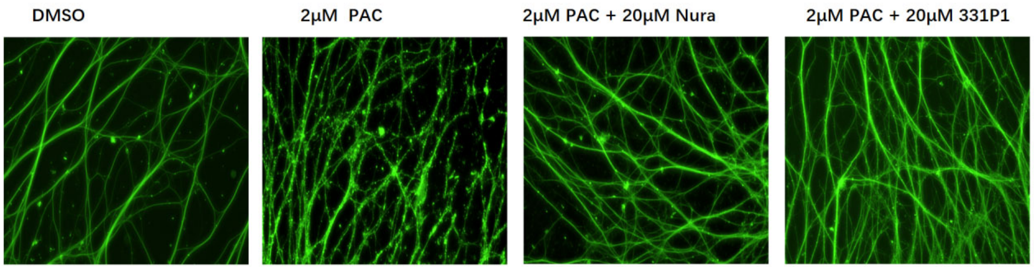

Figure S2. Representative images of Control and SARM1 inhibitor-treated axons at indicated concentration as PAC was added to the axon DRG
